# Multiple *TP53* p.R337H haplotypes and implications for tumor susceptibility

**DOI:** 10.1016/j.xhgg.2023.100244

**Published:** 2023-10-04

**Authors:** Emilia M. Pinto, Cintia Fridman, Bonald C. Figueiredo, Hector Salvador, Manuel R. Teixeira, Carla Pinto, Manuela Pinheiro, Christian P. Kratz, Cinzia Lavarino, Edith A.M. F. Legal, Anh Le, Gregory Kelly, Erika Koeppe, Elena M. Stoffel, Kelsey Breen, Stefanie Hahner, Britta Heinze, Piti Techavichit, Amanda Krause, Tsutomu Ogata, Yasuko Fujisawa, Michael F. Walsh, Huma Q. Rana, Kara N. Maxwell, Judy E. Garber, Carlos Rodriguez-Galindo, Raul C. Ribeiro, Gerard P. Zambetti

**Affiliations:** 1 Department of Pathology, St. Jude Children’s Research Hospital, Memphis, TN, USA; 2 Departamento de Medicina Legal, Bioética, Medicina do Trabalho e Medicina Física e Reabilitação, Faculdade de Medicina da Universidade de São Paulo, São Paulo, Brazil; 3 Instituto de Pesquisa Pelé Pequeno Príncipe, Curitiba, Paraná, Brazil; 4 Pediatric Oncology Department, Sant Joan de Deu Hospital, Barcelona, Spain; 5 Cancer Genetics Group, IPO Porto Research Center (CI-IPOP) / RISE@CI-IPOP (Health Research Network), Porto, Portugal; 6 Department of Laboratory Genetics, Portuguese Oncology Institute of Porto (IPO Porto)/Porto Comprehensive Cancer Center and School of Medicine and Biomedical Sciences (ICBAS), University of Porto, Porto, Portugal; 7 Pediatric Hematology and Oncology, Hannover Medical School, Hannover, Germany; 8 Department of Medicine-Hematology-Oncology, Perelman School of Medicine, University of Pennsylvania, Philadelphia, PA, USA; 9 Department of Internal Medicine, University of Michigan, Ann Arbor, MI, USA; 10 Department of Pediatrics and Medicine, Memorial Sloan Kettering Cancer Center, New York, NY, USA; 11 Department of Medicine I, Division of Endocrinology and Diabetology, University Hospital Wuerzburg, Wuerzburg, Germany; 12 Integrative and Innovative Hematology/Oncology Research Unit, Department of Pediatrics, Faculty of Medicine, Chulalongkorn University, Bangkok, Thailand; 13 Division of Human Genetics, National Health Laboratory Service (NHLS) and Faculty of Health Sciences, School of Pathology, The University of the Witwatersrand, Johannesburg, South Africa; 14 Department of Pediatrics, Hamamatsu University School of Medicine, Hamamatsu, Japan; 15 Division of Cancer Genetics and Prevention, Dana-Farber Cancer Institute, Boston, MA, USA; 16 Department of Global Pediatric Medicine, St. Jude Children’s Research Hospital, Memphis, TN, USA; 17 Department of Oncology, St. Jude Children’s Research Hospital, Memphis, TN, USA

**Keywords:** R337H, founder mutation, haplotype, ancestry, Iberian Peninsula, Brazil, Sephardic Jewish, mitochondrial DNA, Y-STR, cancer predisposition

## Abstract

The germline *TP53* p.R337H mutation is reported as the most common germline *TP53* variant. It exists at a remarkably high frequency in the population of southeast Brazil as founder mutation in two distinct haplotypes with the most frequent co-segregating with the p.E134∗ variant of the *XAF1* tumor suppressor and an increased cancer risk. Founder mutations demonstrate linkage disequilibrium with neighboring genetic polymorphic markers that can be used to identify the founder variant in different geographic regions and diverse populations. We report here a shared haplotype among Brazilian, Portuguese, and Spanish families and the existence of three additional distinct *TP53* p.R337H alleles. Mitochondrial DNA sequencing and Y-STR profiling of Brazilian carriers of the founder *TP53* p.R337H allele reveal an excess of Native American haplogroups in maternal lineages and exclusively European haplogroups in paternal lineages, consistent with communities established through male European settlers with extensive intermarriage with Indigenous women. The identification of founder and independent *TP53* p.R337H alleles underlines the importance for considering the haplotype as a functional unit and the additive effects of constitutive polymorphisms and associated variants in modifier genes that can influence the cancer phenotype.

## Teaser

p.R337H is the most common germline *TP53* variant that exists as a founder and as independent alleles. In this context, the haplotype as a functional unit needs to be considered due to the additive effects of constitutive polymorphisms and associated variants in modifier genes that can influence cancer susceptibility and phenotype.

## Introduction

*TP53* is a tumor suppressor that regulates the cell cycle, senescence, DNA repair, metabolism, and apoptosis in response to DNA damage and other cellular stresses. Given this central role in maintaining cellular homeostasis, it is not surprising that p53 is frequently selected against in human cancer by mutation, thus rendering this tumor suppressor inactive. Although most *TP53* mutations are somatically acquired, individuals who inherit an inactivating *TP53* variant are highly susceptible to developing cancer at an early age with diverse tumor types and multiple malignancies.

The *TP53* p.R337H variant, the most common germline variant in The TP53 Database (formerly the International Agency for Research on Cancer; IARC), was first reported in a pediatric patient of Portuguese ancestry with adrenocortical cancer treated in France.^1^ Previous studies have established p.R337H as a hypomorphic, attenuated mutant allele that confers a highly variable cancer risk ranging from individuals who remain unaffected over their lifetime to those who meet the clinical criteria for classic Li-Fraumeni Syndrome (LFS).^2^^,^^3^^,^^4^ It is now recognized that the *TP53* p.R337H variant is a founder mutation^5^ frequently found throughout south and southeastern Brazil, including the major states of São Paulo and Paraná (total population of 60 million) being detected in one in every 300 individuals.^6^^,^^7^ Carriers of the founder *TP53* p.R337H variant share an identical DNA sequence for the *TP53* locus,^8^ with the majority retaining homology that extends to the telomeric region of chromosome 17 encompassing a nonsense mutation (p.E134∗) in *XAF1* (X-linked inhibitor of apoptosis-associated factor 1).^8^ Previous studies have established XAF1 as a proapoptotic tumor suppressor that functions within a positive autoregulatory loop with *TP53*.^9^ Consistent with these findings, individuals who inherit the compound mutant haplotype (*TP53* p.R337H; *XAF1* p.E134∗) are at an increased risk for cancer in general, sarcomas, and multiple tumors compared with those carriers with only the p.R337H mutation.^8^

Haplotype analysis makes it possible to discriminate between a founder mutation^10^ or a variant resulting from an independent mutational event. This is particularly important in the context of hypomorphic *TP53* variants, as specific polymorphisms can impact p53 expression and function, and thus influence cancer risk.^11^^,^^12^ By integrating genotype, haplotype, and ancestry analyses we have assessed 38 *TP53* p.R337H carriers from South America, North America, Europe, South Africa, and Asia to determine the diversity of the *TP53* p.R337H alleles.

## Materials and methods

### TP53 p.R337H carriers

Genomic DNA samples from 38 unrelated individuals (33 patients with cancer, including 25 females) harboring the *TP53* p.R337H variant from 11 different countries (Brazil, Portugal, Spain, France, Germany, England, United States, Japan, American Samoa, South Africa, and Argentina) were included in this study (Table 1). Personal and family histories of cancer were collected by the genetic counselor or physician at each participating institution. Genetic testing was offered as a *TP53* single gene analysis or as part of a multi-gene hereditary cancer panel (Ambry Genetics, OvaNext; Myriad, Myrisk Hereditary Cancer Test; Illumina, TruSight Hereditary Cancer Panel) with additional genetic findings included in Table 1. The validation cohort included 86 unrelated (documented for at least three generations) and unaffected Brazilian males who carry the *TP53* p.R337H allele. Appropriate institutional informed consent guidelines were followed for all participants or caregivers. This study was approved by local ethics committees and the institutional review board at St. Jude Children’s Research Hospital.

**Table 1:** Geographic origin and demographics of 38 TP53 p.R337H carriers

Country	Primary tumor (age/diagnosis)/Additional tumors	Status	Gender^a^	R337H	E134**∗**	Haplotype
(1) Brazil	ACT (9)	Pediatric/Deceased	M	Pos (H)	Pos (H)	Hap1
(2) Brazil	CPC (6)	Pediatric	M	Pos (H)	Pos (H)	Hap1
(3) USA/Brazil	ACT (3)	Pediatric	M	Pos	Neg	Hap2
(4) USA/Brazil	ACT (1)	Pediatric	F	Pos	Pos	Hap1
(5) Brazil	ACT (3)	Pediatric	M	Pos	Pos	Hap1
(6) Brazil	ACT (2)	Pediatric/Deceased	F	Pos	Pos	Hap1
(7) Argentina	ACT (4)	Pediatric/Deceased	F	Pos	Pos	Hap1
(8) Brazil	ACT (11)	Pediatric	F	Pos	Pos	Hap1
(9) Argentina	ACT (9)	Pediatric	F	Pos	Neg	Hap3
(10) Spain	ACT (3)	Pediatric	M	Pos	Pos	Hap1
(11) Spain	ACT (2)	Pediatric	M	Pos	Pos	Hap1
(12) Spain	Unaffected A&W (19)	Adult	M	Pos	Pos	Hap1
(13) Portugal	ACT (4)	Pediatric	F	Pos	Neg	Hap2
(14) Portugal/Brazil	ACT (4)	Pediatric	F	Pos	Pos	Hap1
(15) Portugal	Liposarcoma (40)	Adult	F	Pos	Pos	Hap1
(16) Portugal	Gastric (62)	Adult	M	Pos	Pos	Hap1
(17) Portugal	Breast Her2+ (40)	Adult	F	Pos	Pos	Hap1
(18) USA/Brazil	ACT (34)	Adult	M	Pos	Neg	Hap2
(19) USA	Breast (41)	Adult	F	Pos	Neg	Hap4
(20) USA	Breast (39), Thyroid (57), Leiomyosarcoma (62)	Adult	F	Pos	Neg	Hap4
(21) USA	Thyroid (27), Breast (36), Lung (59), Melanoma (60)	Adult/Deceased	F	Pos	Neg	Hap4
(22) USA	Unclassified spindle cell sarcoma (27), Breast (29), Sarcoma (33)	Adult	F	Pos	Neg	Hap4
(23) USA	Basal cell carcinoma (53)	Adult	F	Pos	Neg	Hap3
(24) USA	Unaffected A&W (39)	Adult	M	Pos	Neg	Hap3
(25) USA	Breast (38)	Adult	F	Pos	Pos	Hap1
(26) USA	Breast (45), Thyroid (55)	Adult	F	Pos	Neg	Hap4
(27) USA	Breast (46), Breast (49), Head and neck (59), STS (61)	Adult	F	Pos	Neg	Hap4
(28) USA/Brazil	Breast (45), Low grade mucinous appendiceal carcinoma (47)	Adult	F	Pos	Neg	Hap2
(29) France	Breast (64), Endometrial (77)	Adult	F	Pos	Neg	Hap3
(30) Germany	Colon (65), ACT (71), Prostate (74)	Adult/Deceased	M	Pos	Neg	Hap3
(31) England	Unaffected A&W (45)	Adult	F	Pos	Neg	Hap3
(32) Japan/Brazil	ACT (2)	Pediatric	M	Pos	Pos	Hap1
(33) Samoa	ACT (9)	Pediatric	F	Pos (de novo)	Neg	Hap5
(34) Germany	Tubal carcinoma (54), Peritoneal carcinomatosis (58)	Adult	F	Pos	Neg	Hap3
(35) Germany	Unaffected A&W (29)	Adult	F	Pos	Neg	Hap3
(36) Germany	Unaffected A&W (30)	Adult	M	Pos	Neg	Hap3
(37) Germany	Gastric (62)	Adult	F	Pos	Neg	Hap3
(38) South Africa	ACT (5)	Pediatric	F	Pos	Neg	ND

#15, 16, 17, 19, 20, 21, 25, 26, 28 and 29 are *BRCA1/2* negative by genetic panel testing.

#22 is positive for *BRCA1* c.4065_4068del(TCAA) inherited from father.

A&W, alive and well (no current cancer); ACT, adrenocortical tumor; CPC, choroid plexus carcinoma; F, female; H, homozygous; M, male; ND, not determined; STS, soft tissue sarcoma.

a Gender defined by biological attributes.

### Haplotype determination of recurrent TP53 p.R337H alleles

Genomic DNA was isolated and used to determine *TP53* sequence and the *XAF1* p.E134∗ variant status as previously reported.^8^ Additionally, fluorescent-labelled PCR products for 10 polymorphic microsatellite markers located within (VNTRp53) or telomeric to the *TP53* locus (Table S1) were genotyped by using the ABI 3500 sequencer (ThermoFisher Scientific) and sizes assigned to the different fragments using GeneMapper v 6.0 (ThermoFisher Scientific).^8^^,^^13^ Haplotypes were determined by segregation analysis of genomic DNA from parents and descendants, loss of heterozygosity in tumor samples, or inferred.

### Databases

Information for the *TP53* p.R337H variants was retrieved from The TP53 Database (https://tp53.isb-cgc.org/; resource for more than 30,000 somatic and germline *TP53* variants in human cancer); ClinVar (https://www.ncbi.nlm.nih.gov/clinvar/; public archive for human variants and associated clinical phenotypes); gnomAD (https://gnomad.broadinstitute.org/; repository for more than 140,000 exome and whole genome sequences selected from human disease-specific and population genetic studies); Global Biobank Engine (https://biobankengine.stanford.edu/; summary of genotype-phenotype correlations in large biobank cohorts representing 750,000 individuals); ABraOM (abraom.ib.usp.br; archive of exome and whole genome sequences from >1,000 unrelated, elderly (>70 years old) individuals from São Paulo, Brazil); the Precision Oncology Knowledge Base (OncoKB; https://www.oncokb.org/; housed by Memorial Sloan Kettering Cancer Center representing more than 7,000 genetic alterations in 134 cancer types); and the Catalog of Somatic Mutations in Cancer (COSMIC; https://cancer.sanger.ac.uk/cosmic).

### Mitochondrial DNA control region

The nucleotide sequence of mitochondrial DNA (mtDNA) hypervariable segment 1 (HVI; between nucleotide positions 16024 and 16365), segment 2 (HV2; between nucleotide positions 73 and 340), and segment 3 (HV3, covering the position 438 to 574) of mtDNA control region was determined for 37 of 38 probands, and available family members (n = 26). We have also determined the mtDNA haplotype for all Brazilian male individuals harboring the p.R337H allele (validation cohort) inherited from their mother (n = 45).

Amplification of the mtDNA control region was performed by using L15781 5′-CCCTTTTACCATCATTGGACA-3′ and H727 5′-AGGGTGAACTCACTGGAACG-3′ primers. Amplified segments were sequenced by using L15781, H16478, L109, H408, and H727 primers as previously described.^14^ Resulting sequences were compared with the *Homo sapiens* mitochondrion, complete genome (NCBI reference sequence: NC_012920.1), known as revised Cambridge Reference Sequence (rCRS). Haplogrep software (https://haplogrep.i-med.ac.at/), EMPOP (https://empop.online/), and visual inspection analyses were used to define the mtDNA haplogroups.

### Y chromosome markers

A set of 23 Y-STRs (short tandem repeats) was analyzed for 13 unrelated male p.R337H carriers and four first-degree male relatives in the main cohort of 38 individuals using the PowerPlex Y23 System (Promega) as described by the manufacturer. Y-STR haplotypes were also determined for Brazilian male individuals harboring the p.R337H allele (validation cohort) that was paternally inherited (n = 41). Alleles were separated and detected on an ABI 3500 sequencer (ThermoFisher Scientific) and sizes were assigned to the different fragments using GeneMapper v 6.0 (ThermoFisher Scientific). The alleles were named according to the number of repeated units, based on the sequenced allelic ladder following International Society for Forensic Genetics recommendations.^15^^,^^16^ The classification of Y chromosome haplogroup was done using the Haplogroup Predictor program FTDNA 2.0 (http://www.hprg.com/hapest5/index.html) and Y chromosome Haplotype Reference Database (https://yhrd.org/search).

## Results

### Haplotypes of TP53 p.R337H carriers

**Figure 1:**
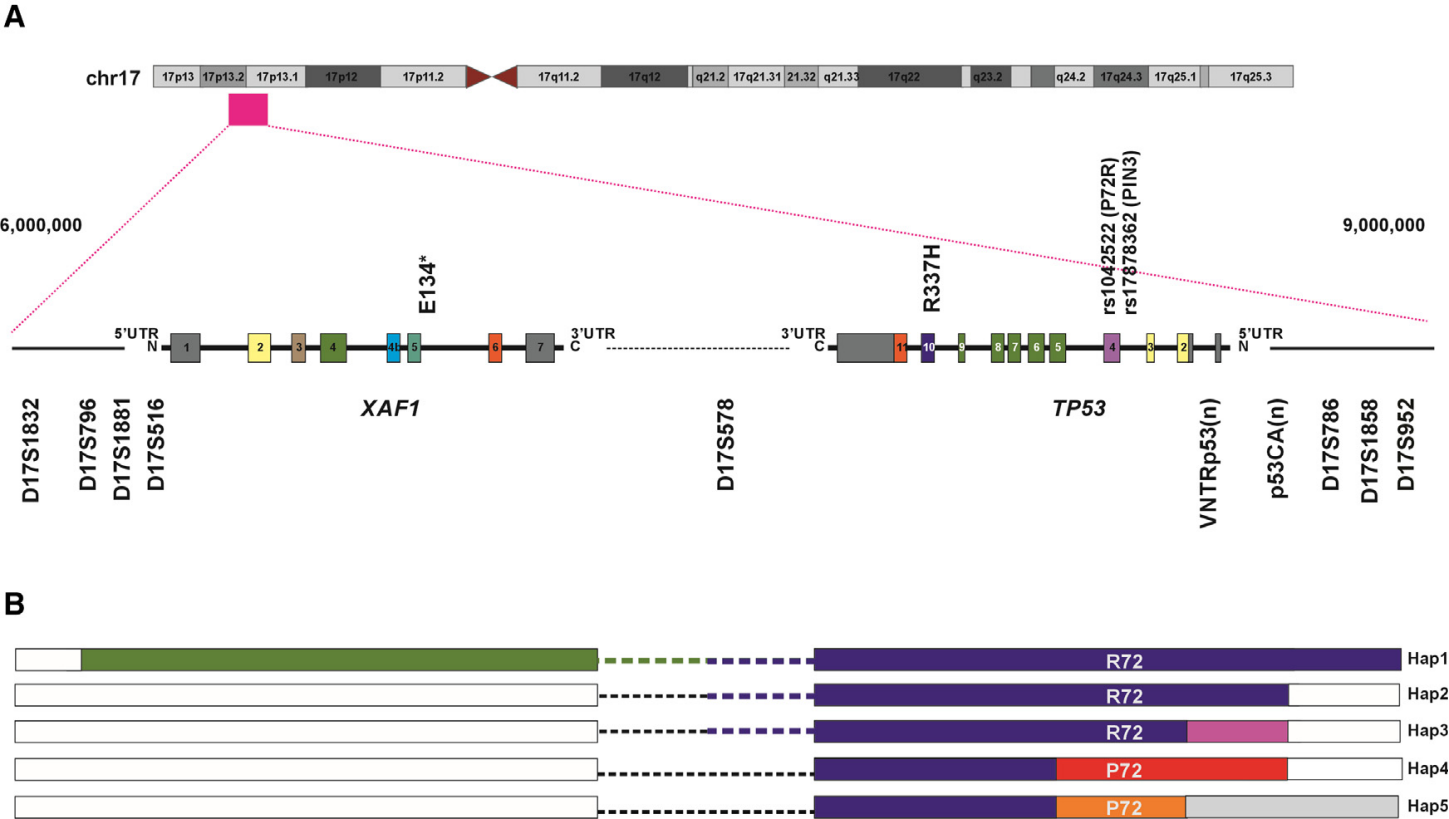
Diagram of five distinct *TP53* p.R337H alleles (A) Five distinct haplotypes were identified based on *TP53* internal polymorphisms and microsatellite markers on chromosome 17p13. (B) Identical DNA sequences shared among the five haplotypes are identified by solid blue bars and blue dashed lines. Polymorphic markers distinguishing the five haplotypes are highlighted in purple [Hap3, VNTRp53(n) and p53CA(n)], red [Hap4 P72R, PIN3, VNTRp53(n) and p53CA(n)], orange (Hap5, de novo, P72/A2), and green (Hap1, DNA sequence spanning D17S796 to D17S952 containing *XAF1* p.E134∗ variant) solid bars.

*TP53* sequence and chromosome 17 polymorphic marker analyses of genomic DNA from 38 probands (Table 1) who carry the *TP53* p.R337H variant revealed multiple distinct haplotypes (Figure 1; Table S2). *TP53* p.R337H alleles were distinguished by the common polymorphisms at codon 72 (rs1042522) encoding proline (P72) or arginine (R72), and PIN3 (rs17878362) containing one (A1) or two copies (A2) of a 16-base pair sequence in intron 3. In this cohort of *TP53* p.R337H carriers, R72 co-segregates with A1 (R72/A1) and P72 co-segregates with A2 (P72/A2). Excluding one undetermined case (#38), carriers with R72/A1 were more frequently observed (n = 30) compared with P72/A2 genotype (n = 7), establishing two distinct *TP53* p.R337H alleles. Extending the analysis to include the polymorphic marker VNTRp53, a pentanucleotide (AAAAT)_n_ repeat within the 6.1-kb-long *TP53* intron 1,^17^^,^^18^ the p53CA(n) polymorphic marker,^19^ and the *XAF1* p.E134∗ variant,^8^ we delineated five *TP53* p.R337H haplotypes (Figure 1; Table S2).

The most common *TP53* p.R337H haplotype (hereafter Hap1) (n = 16 carriers; two homozygous) spanned from D17S796 (chr17:6,251,522–6,251,775; GRCh37/hg19) to D17S952 (chr17:9,113,650–9,113,834; GRCh37/hg19) and contains the *XAF1* p.E134∗ variant. An identical, but shorter, p.R337H haplotype (Hap2), that lacks *XAF1* p.E134∗, extended from D17S578 (chr17:6,823,880; GRCh37/hg19) to the marker p53(CA)_n_ (chr17:7,617,440 -7,617,490; GRCh37/hg19) (n = 4 individuals) (Figure 1; Table S2). These two *TP53* p.R337H haplotypes (Hap1 and Hap2), which comprise the R72/A1 polymorphisms and consistent alleles for VNTRp53(n) and p53(CA)_n_ have been characterized as a founder p.R337H variant, widespread in Brazil.^5^^,^^8^ Identical Hap1 (n = 7) and Hap2 haplotypes (n = 1) were also identified in the eight unrelated families from Spain and Portugal (Table 1; Table S2).

A third *TP53* p.R337H haplotype containing the R72/A1 polymorphisms but distinct from Hap1 and Hap2 based on allelic differences in the VNTRp53(n) and p53CA_(n)_ polymorphic markers (Hap3) was observed in 10 probands from Europe and the United States (Figure 1; Table S2). A *TP53* p.R337H allele with P72/A2 (Hap4) was observed in six individuals from the United States. A documented *de novo TP53* p.R337H variant with P72/A2 (Hap5) was identified in a pediatric ACT patient from an American Samoa family (Table 1).^20^ In addition, a heterozygous *TP53* p.R337H proband with ACT from South Africa (#38)^21^ was identified, but the haplotype, and whether it was inherited or *de novo*, was not determined due to unavailability of parental DNA or tumor samples. Nonetheless, microsatellite analysis excluded this germline variant as being Hap 1 or Hap2.

**Figure 2:**
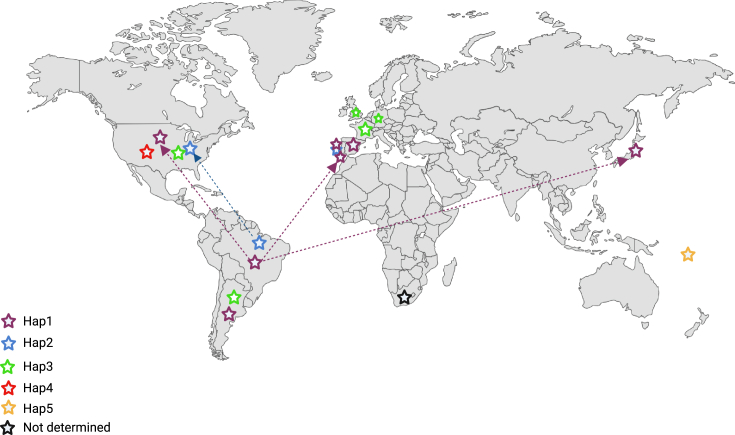
World map showing the distribution of *TP53* p.R337H alleles and their constitutive haplotypes Known migration routes of carriers from Brazil to the United States, Portugal, and Japan are highlighted by purple (Hap1) and blue (Hap2) arrows with dotted lines.

The country of birth and migration status when known for each proband was annotated (Figure 2; Table 1). Carriers of Hap1 (n = 3) and Hap2 (n = 2) identified in the United States and Japan are known to have immigrated from Brazil (Table 1). However, seven of the eight families from the Iberian Peninsula (Spain and Portugal) report no connection to Brazil.

### Frequency of reported TP53 p.R377H alleles in public databases

Of the 4,453 reported germline mutations (n = 1,655 families) in The TP53 Database (R20, July 2019 version; https://tp53.isb-cgc.org/), p.R337H was observed in 305 individuals (n = 117 families) representing 240 patients with cancer and 65 carriers unaffected by cancer. This database includes cases from Brazil (n = 282; 101 families), France (n = 7; 6 families), Germany (n = 4; 2 families), Japan (n = 5; 1 family), China (n = 1), Portugal (n = 1), and South Africa (n = 1) and an additional four cases whose country of origin was not identified. ClinVar (https://www.ncbi.nlm.nih.gov/clinvar/) also lists 21 entries for the *TP53* p.R337H allele (variation ID 12379), with 18 being classified as pathogenic and three as likely pathogenic. Of note, p.R337H cases are often presumed to be the founder variant without recognizing differences in *TP53* sequences (e.g., constitutive internal polymorphisms) or associated haplotypes.^22^^,^^23^

*TP53* p.R337H was also reported as a somatic mutation in (1) The TP53 Database (n = 4), (2) The OncoKB database (n = 3), and (3) The COSMIC database (COSM43882, COSM4173039; n = 22). However, the germline status of these patients with p.R337H-positive tumors was not established. The *TP53* p.R337H variant was not reported at Global Biobank Engine and ABraOM databases. However, it was observed in the Genome Aggregation Database (gnomAD) in three female individuals (two cancer patients, one unaffected by cancer), two identified as Latino/Admixed American individuals.

Interestingly, the *TP53* p.R337H variant has also been observed in two ancient genomes (European Nucleotide Archive; ENA Browser [www.ebi.ac.uk]), one carrier from Mongolia and the other from Italy. Whether these ancient alleles are fixed in the human lineage or independent events was not determined.^24^

### Family histories of cancer

The cancer history for the 38 p.R337H carriers in this study was collected (Table 1). All six Hap4 probands developed breast cancer at an early age (Table 1; range from 29 to 46 years old) with five developing multiple LFS core tumor types. Except for one proband (#22), all cases tested negative for *BRCA1/2* mutations.

The cancer phenotype for Hap3 probands (n = 10) appears to be milder than Hap4, with four adults currently unaffected by cancer and the remaining six carriers having developed non-LFS core tumors with late onset (Table 1). Hap3 proband #24 (39 years old), presently unaffected by cancer, was self-reported to be of Ashkenazi Jewish descendant with no remarkable family history of cancer, except for a nephew with a diagnosis of choroid plexus carcinoma at age 2. Excluding two carriers who developed childhood ACT (proband #37, *de novo* p.R337H variant; proband #38, *de novo* or inherited not determined), nearly all pediatric tumors in the current cohort (n = 14) were associated with the Hap1 and Hap2 founder p.R337H mutation (n = 13; 93%).

### Ancestry of the TP53 p.R337H variants

The maternal genetic composition of carriers of the *TP53* p.R337H variant was investigated by characterizing the mitochondrial control region sequences (HV1-3) from the 37 probands (Table S3; proband #30 DNA unavailable). Altogether, *TP53* p.R337H carriers fall into four major mtDNA haplogroups with the predominance being of European descent (H, J, K, R, U, V, X; n = 23, 61%) followed by Native American (A–D; n = 6, 16%), African (L; n = 6, 16%), and Asian descent (M7c; n = 2, 5%). The paternal composition was examined by genotyping 23 Y-STRs from 13 male probands in this cohort. These results indicate that the paternal lineage of carriers is associated with the following European haplogroups: R1b (n = 6), J1 (n = 3), I2a1 (n = 2), G2a (n = 1), and E1b1b (n = 1) (Table S4).

Ancestry of Hap 1 (n = 65) and Hap2 (n = 21) was analyzed in a validation cohort of 86 independent Brazilian male *TP53* p.R337H carriers who were identified through a general population neonatal screening program (Tables S3 and S4).^6^ This cohort provided the opportunity to determine Y-STR profiling and mitochondrial DNA sequence analysis of carriers with paternal and maternal inheritance of *TP53* p.R337H, respectively. Of those male Brazilian carriers who inherited the p.R337H variant from their fathers (n = 41; 33 Hap1 and 8 Hap2), all Y-STR haplotypes were found to be associated with European/Eurasian descent, primarily the major haplogroup R (n = 23, 56%; in particular R1b, n = 22) followed by E (n = 7; 17%), I (n = 5; 12%), J (n = 3; 7%), L (n = 2; 5%), and Q (n = 1; 2%) (Table S4). For those male Brazilian carriers who inherited the p.R337H variant from their mothers (n = 45; 32 Hap1 and 13 Hap2), mitochondrial haplotypes were predominantly associated with Native American ancestry (haplogroups A–D; n = 33, 73%), followed by European/Eurasia (n = 9, 20%) and African (n = 3, 7%) haplogroups (Table S3). Although the 86 male individuals selected for this study were recruited from different families and documented for at least three generations, we have identified relatedness in 15 cases (17%) based on shared mtDNA sequences (n = 11; A–D haplogroups) or Y-STR (n = 4; I2b1 and E1b1a haplogroups), consistent with a founder effect^5^^,^^8^ (Tables S3–S5).

## Discussion

In the present study we have identified five distinct *TP53* p.R337H haplotypes, with two (Hap1 and Hap2) sharing an identical Caucasian founder allele widespread in the Brazilian population.^5^^,^^8^ The *TP53* p.R337H founder variant co-segregates with *XAF1* p.E134∗ in the majority of Brazilian cases (Hap1) and as a single mutation (Hap2).^8^ Hap1 was also observed in the majority of cases from Spain and Portugal. The remaining three *TP53* p.R337H haplotypes (Hap3-5) that have been identified outside of Brazil are distinct from the founder allele based on internal polymorphisms and polymorphic markers along chromosome 17p.

The p.R337H variant has been functionally characterized by studies *in vitro* and *in vivo* as hypomorphic,^2^^,^^25^^,^^26^^,^^27^ with individuals exibiting a wide range of cancer phenotypes. In many instances, however, the p.R337H variant has been classified as pathogenic and carriers, independent of haplotype and other possible pathogenic variants, are considered to be associated with classic LFS.^28^

The *TP53* p.R337H haplotypes identified here diverge in internal *TP53* polymorphisms, such as codon 72 (Pro72Arg) and PIN3 (intron 3), reported to affect p53 expression and function.^11^^,^^12^ Notably, codon 72 is part of the p53 proline-rich motif that is critical for interactions with MDM-2,^29^ ASPPs,^30^ XAF1,^9^ NF-κB,^31^ and proteins containing the SH3 domain.^32^ Specifically, the R72 variant is more active at stimulating cellular apoptosis and suppressing cellular transformation.^33^ In addition, the PIN3 variant (A2) has been associated with decreased p53 expression^34^ and several case-control studies have reported an increased risk of various cancer types associated with the A2 allele in Caucasians, with the most consistent association reported for breast and colorectal cancers.^35^ It is well established that the combination of specific variants in *cis* can potentially modify the penetrance of coding variants,^36^ with these variants exhibiting much smaller effects individually but with additive haplotypic effects. Phenotypic variability associated with another variant *in cis* is exemplified by the presence of *XAF1* p.E134∗ in a subset of *TP53* p.R337H carriers (Hap1) that are at higher risk of cancer in general, sarcomas, and multiple tumors compared with those with *TP53* p.R337H alone (Hap2).^8^

Although the number of carriers of Hap3 and Hap4 were not sufficient to establish a clear haplotype-cancer phenotype association, we did observe a pattern whereby Hap4 carriers more closely resembled classical LFS, including breast cancers, sarcomas, and multiple primary tumors. In contrast, Hap3 carriers present generally with tumors not seen typically in LFS and at late onset with incomplete penetrance, suggestive of a lower risk of cancer.

To determine the ancestry and occurrence of *TP53* p.R337H variant within the framework of the history of Brazil, our analysis of mitochondrial haplogroups of carriers defined an excess of Native American haplogroups (A–D; 73%) in maternal lineages. Although many native groups in Brazil have vanished, particularly in southeast regions,^37^ our observed 73% of Brazilian p.R337H carriers with the Native American matrilineal lineages is in clear contrast with present day Brazilians in the same region that still carry the genetic imprinting of early colonization phase but with much more uniform distribution: 39% European, 33% Amerindian, and 28% African lineages.^37^ Y-STR profiling of carriers from the paternal lineage revealed exclusively European haplogroups, particularly R1b. This composition indicates a remarkable signature among communities established by European male settlers with extensive intermarriage with Indigenous women.^38^ We postulate that Iberian Peninsula Europeans introduced the *TP53* p.R337H variant in the Brazilian population in early colonial times. Our hypothesis is based on the knowledge that the European-Amerindian admixture started soon after the arrival of the first colonizers.^38^ In addition, we observed a high frequency of Y-STR haplogroup R1b in Brazilian carriers as well as in *TP53* p.R337H carriers from Portugal and Spain. It is well established that R1b haplogroup is the most common Y chromosome branch of Atlantic Europe, the most frequent haplogroup in the Iberian Peninsula and with great representation among Sephardic Jews.^39^ Of note, our Spanish p.R337H carriers are from Extremadura, a Spanish border region adjacent to Portugal, with large Sephardic communities and a prominent source for migrants to the Americas during the 16th century.^40^

Although we cannot determine whether the p.R337H founder allele first arose within the context of Hap1 or Hap2, we have observed the uncoupling of *XAF1* p.E134∗ and *TP53* p.R337H in several Hap1 Brazilian families.^8^ These findings suggest that the region separating *XAF1* and *TP53* on chromosome 17 is susceptible to recombination and that gene conversion could promote haplotype diversity. These findings also highlight the importance of determining the haplotype of this hypomorphic mutation in each carrier even when occurring within the same family.^8^

In conclusion, we have identified five distinct *TP53* p.R337H haplotypes that differ in specific intragenic and extragenic polymorphisms, as well as associated modifiers. Given the hypomorphic nature of p.R337H, in contrast to pathogenic DNA binding domain *TP53* mutants, its tumor suppressor activity can be significantly influenced by associated polymorphisms and variants. One such example is the p.R337H founder mutation that is common throughout southern Brazil, but in the context of two haplotypes that differ in the status of *XAF1* and tumor susceptibility (Hap1 and Hap2).^8^^,^^41^^,^^42^ As shown here, additional non-founder independent p.R337H alleles (Hap3-5) have been identified that also likely influence cancer risk. Therefore, genetic counselors and health care providers must be aware of these distinct p.R337H haplotypes and the potential implications of their associated differences on p53 expression, structure, and function, and consequently cancer risk.
